# Are algal genes in nonphotosynthetic protists evidence of historical plastid endosymbioses?

**DOI:** 10.1186/1471-2164-10-484

**Published:** 2009-10-20

**Authors:** John W Stiller, Jinling Huang, Qin Ding, Jing Tian, Carol Goodwillie

**Affiliations:** 1 Department of Biology, East Carolina University, Greenville, USA; 2 Department of Computer Science, East Carolina University, Greenville, USA

## Abstract

**Background:**

How photosynthetic organelles, or plastids, were acquired by diverse eukaryotes is among the most hotly debated topics in broad scale eukaryotic evolution. The history of plastid endosymbioses commonly is interpreted under the "chromalveolate" hypothesis, which requires numerous plastid losses from certain heterotrophic groups that now are entirely aplastidic. In this context, discoveries of putatively algal genes in plastid-lacking protists have been cited as evidence of gene transfer from a photosynthetic endosymbiont that subsequently was lost completely. Here we examine this evidence, as it pertains to the chromalveolate hypothesis, through genome-level statistical analyses of similarity scores from queries with two diatoms, *Phaeodactylum tricornutum *and *Thalassiosira pseudonana*, and two aplastidic sister taxa, *Phytophthora ramorum *and *P. sojae*.

**Results:**

Contingency tests of specific predictions of the chromalveolate model find no evidence for an unusual red algal contribution to *Phytophthora *genomes, nor that putative cyanobacterial sequences that are present entered these genomes through a red algal endosymbiosis. Examination of genes unrelated to plastid function provide extraordinarily significant support for both of these predictions in diatoms, the control group where a red endosymbiosis is known to have occurred, but none of that support is present in genes specifically conserved between diatoms and oomycetes. In addition, we uncovered a strong association between overall sequence similarities among taxa and relative sizes of genomic data sets in numbers of genes.

**Conclusion:**

Signal from "algal" genes in oomycete genomes is inconsistent with the chromalveolate hypothesis, and better explained by alternative models of sequence and genome evolution. Combined with the numerous sources of intragenomic phylogenetic conflict characterized previously, our results underscore the potential to be mislead by *a posteriori *interpretations of variable phylogenetic signals contained in complex genome-level data. They argue strongly for explicit testing of the different *a priori *assumptions inherent in competing evolutionary hypotheses.

## Background

Completely sequenced eukaryotic genomes represent large and complex data sets, and phylogenomic investigations generally uncover numerous conflicts among individual gene phylogenies [1,2]. When a given gene produces a phylogeny with strong support for an alternative relationship to what generally is accepted, it is viewed as a likely candidate for horizontal (or lateral) gene transfer (HGT) [3]. Multiple genes from a given genome supporting the same discrepant relationship are interpreted as evidence of correlated HGT, stemming from an historical endosymbiosis in the organism's ancestors [4-6].

Genomes of all photosynthetic eukaryotes contain numerous sequences acquired via HGT from cyanbacterial ancestors of plastids. Interpreting cases of endosymbiotic gene transfer (EGT) and endosymbiotic gene replacement ("EGR" - when the endosymbiont's gene replaces a previously existing homolog) can be relatively straightforward in organisms like green plants and red algae that harbor "primary plastids" (the endosymbiont was a cyanobacterium). Some algal groups, however, are products of secondary or higher-order endosymbioses, meaning they adopted a eukaryotic endosymbiont along with its pre-existing plastid. In these cases, the host genome acquires not only cyanobacterial genes via EGT and EGR, but also eukaryotic sequences from the nucleus of the endosymbiont [7].

Large-scale impacts from EGT and EGR have important implications for understanding eukaryotic relationships and, in particular, whether and how plastids have moved among major lineages [7-9]. For example, they could provide evidence of a transient endosymbiosis in taxa for which there is no current cytological indication of an active or vestigial plastid. Consequently, a number of efforts have been made to look for evidence of EGT/EGR from a photosynthetic endosymbiont that could have been lost from parasitic and heterotrophic relatives of various algal groups [10-12].

The completed genomes of the oomycetes *Phytophthora ramorum *and *P. sojae *were found to contain multiple genes that imply phylogenetic affiliations with red algae and cyanobacteria [13]. The presence of these genes has been interpreted widely as support for the chromalveolate model [7,8,12,13], which argues that algal groups (ochrophytes, cryptophytes, haptophytes, dinoflagellates, apicomplexans) with red algal-derived plastids trace to a common photosynthetic ancestor [14]. During the establishment of this endosymbiont and its transition to a fully integrated organelle, the host cell nucleus would have accumulated some unknown fraction of red algal and cyanobacterial genes via EGT and EGR. The model further stipulates that this "red" plastid subsequently was inherited by genealogical descent, meaning that extant, aplastidic relatives of these algae must have lost the organelle along the way. Thus, the presence of "algal" genes in *Phytophthora *genomes is cited as key evidence that non-photosynthetic heterokonts (stramenopiles) once harbored the same plastid now present in their close relatives, the ochrophytes (e.g. diatoms and brown algae).

"Algal" genes also have been found in several other aplastidic members of the "Chromalveolata" and, likewise interpreted as potential support for this broader model of plastid and organismal evolution [5,11,12]. Such *a posteriori *results from genome-level data mining are difficult to interpret, however, because they do not address whether the amount of aberrant phylogenetic signal found is significantly greater than expected from null or alternative models. Persistently discordant gene phylogenies have a number of possible explanations; they are consistent with directional phylogenetic artifacts [15-17], horizontal gene transfers associated with feeding preferences or other symbiotic associations [8,18], and alternative models of endosymbiotic plastid transfer [19-21]. Therefore, it is critical to test whether algal genes in aplastidic protists explicitly support a given evolutionary model such as the Chromalveolata. It is particularly important that tests be structured to include appropriate controls that demonstrate observed phylogenetic affinities are not simply an expected outcome from intragenomic co-variation in tree-building signal.

In genome-level surveys, comparisons of raw similarity scores are the most sensitive method for detecting cases of gene transfer [22], and provide rapid, quantitative and reproducible data for identifying and ranking HGT candidates [23]. To improve selectivity, individual genes extracted by genome-wide BLAST surveys and/or automated phylogenetic pipelines, generally are examined more thoroughly using broader sampling and model-based phylogenetic approaches [8,24]. These more rigorous phylogenetic treatments remain computationally intractable on gene-by-gene basis, particularly across four large eukaryotic genomes as we investigate here (see below). Moreover, it is unclear how the relative strengths of cumulative phylogenetic signals favoring competing hypothesis would be assessed statistically. Because of these limitations, most comparative genomic investigations [25], including a number with important phylogenomic implications [26-28], have been based on recognized correlations between similarities in blast scores and phylogenetic signal [29]. This well-demonstrated relationship is an explicit assumption of automated pipelines used to identify likely HGT/EGT candidates from whole genomes for more detailed phylogenetic analyses [8,22,30]. Therefore, we analyzed the relative strength of support for top blastp hits to designated eukaryotic groups as a statistical proxy for aggregate phylogenetic signal. We also employed clear positive controls that validate the use of this methodology.

**Figure 1:**
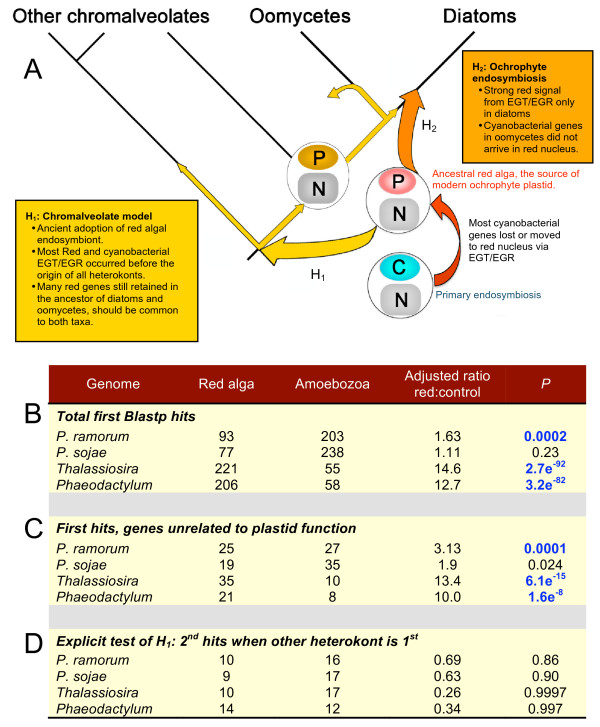
**Specific tests of the chromalveolate versus ochrophyte-specific models**. A. The chromalveolate model assumes the plastid present in modern ochrophytes was adopted as a red algal endosymbiont in the distant ancestor of all chromalveolate taxa, meaning this plastid was lost from oomycetes after they diverged from ochrophytes. Thus, the model (H_1_: yellow box and arrows) makes explicit and testable predictions. In contrast, an ochrophyte-specific origin of the diatom plastid (H_2_: orange box and arrow) makes alternative predictions. B. Fisher exact tests for excess gene signal in heterokont genomes from red algae versus the amoebozoan control. When adjusted for genome size, there are proportionally more first hits to red algae than to amoebozoans in *P. ramorum *but not in *P. sojae*. Both diatom genomes display highly significant excess signal from red algal genes. C. The same tests on only those genes present in all eukaryotic groups, showing the strong red signal in diatoms is not simply from plastid-related genes. D. Same tests (on genes present in all eukaryotic groups) on second hits when the first hit is to the sister heterokont. There is no indication of an excess red algal signal in either oomycete genome. More significantly, the extraordinary signal for a red contribution to the diatom genomes disappears in gene specifically conserved between oomycetes and diatoms. Significant results after adjustments for multiple tests in B-D are shown in blue bold text.

We identified three explicit assumptions of the chromalveolate model that can be tested directly, to determine whether they are supported over null or alternative hypotheses (Figure 1A). These are, 1) if putative cyanobacterial genes in oomycete genomes are to be considered evidence of a red algal endosymbiosis (given that most already resided in the nuclear genome of the engulfed rhodophyte), then, as a group, they should show greater affinity to red algal genomes than do genes with stronger similarities to other bacterial groups; 2) the signal from red algal genes should be proportionally stronger in oomycete genomes than signal from control eukaryotic taxa thought to be unrelated to heterkonts, either phylogenetically or through endosymbiosis, and 3) because of the relative antiquity of the presumed chromalveolate endosymbiosis and associated EGT/EGR, signal from red algal genes specifically unrelated to plastid function should be shared between oomycetes and diatoms. To determine whether putative "red algal" and "cyanobacterial" genes in *Phytophthora *genomes provide support for the chromalveolate model, we applied statistical tests of these clear *a priori *expectations to comparative results against defined control groups.

## Results and Discussion

### Controlling for small size and unusual evolutionary rates of available red algal genomes

We queried a large database of eukaryotic and bacterial genomes with genes from two species of *Phytophthora*, and from two diatoms as positive controls for effects from the known red algal endosymbiosis in ochrophytes. Our initial analyses of overall similarities from BLASTP searches, querying with oomycete genomes, did not suggest support for the chromalveolate model. Although there are expectedly large numbers of top hits to phylogenetically related diatom genomes (*P. ramorum*: 1493/*P. sojae*: 1391), there were even more to animals (1549/1510) and green plants (2861/3135). Conversely, among all eukaryotic groups, the fewest number of top hits were to red algal genes (93/77); this was even fewer than to three of four bacterial groups.

These raw measures of similarity also revealed a conspicuous trend in the data. There was a positive association between the number of top BLASTP hits to a given group and the total number of genes present in the data set queried; that is, the more total genes present in the group searched, the greater the number of first hits returned. Because they could bias statistical comparisons, particularly given the relatively small size of the red algal test group (Additional file 1), we operated under the assumption that these correlations reflect sampling artefacts and controlled for them in all direct tests of the chromalveolate hypothesis. First, we chose as our control the aplastidic eukaryotic group (Amoebozoa) with the data set closest in size and most similar to red algae with respect to shape of similarity score distributions (see below). Second, we designed all contingency analyses to account for differences in database sizes. Finally, and most significantly, we used a positive control to demonstrate that a red algal signal is detectable in non-plastid-related genes when a known endosymbiosis has occurred, despite the small sizes and unusual characteristics of the available rhodophyte genomes.

### Do cyanobacterial genes in oomycetes support the chromalveolate model?

Fisher exact tests of the distribution of first hits to bacterial groups queried with *Phytophthora *genes suggest that cyanobacteria have made a somewhat greater than expected contribution to oomycete genomes (Table 1). In four of the six comparisons there are relatively more hits to cyanobacterial genomes than to control groups, although no differences are significant when thresholds are adjusted for multiple tests [31]. By comparison, Fisher exact tests of top hits to bacterial groups by sequences from the two diatom genomes yield a highly significant prevalence of cyanobacterial signal in five of six cases (Table 2).

**Table 1:** Fisher exact tests for top hits to cyanobacteria relative to bacterial control groups.

**Group**	**First hits**	**Adjusted ratio^1 ^cyano:control**	***P*^2^**
*P. ramorum *as query			

Cyanobacteria	155		

Firmicutes	69	1.27	0.06

Actinobacteria	123	1.13	0.17

Proteobacteria	166	0.82	0.96

*P. sojae *as query			

Cyanobacteria	155		

Firmicutes	62	1.42	0.01

Actinobacteria	95	1.46	0.002

Proteobacteria	166	0.82	0.96

*Thalassiosira *as query			

Cyanobacteria	263		

Firmicutes	51	2.92	**5.0e-15**

Actinobacteria	45	5.27	**5.8e-34**

Proteobacteria	162	1.43	**0.0003**

*Phaeodactylum *as query			

Cyanobacteria	231		

Firmicutes	46	2.85	**5.8e-13**

Actinobacteria	63	3.29	**3.3e-20**

Proteobacteria	166	1.23	0.044

^1^Ratios of top hits are based on scaled proportions of total dataset sizes.

^2^*P *values that are significant after adjustment for multiple tests are shown in bold.

**Table 2:** Fisher exact tests for signal from cyanobacterial genes suggesting a red ancestry.

**Group**	**1^st ^hits, reds not 2^nd^**	**1^st ^hits, reds 2^nd^**	**Scaled ratio cyano:other^1^**	***P*^2^**
*P. ramorum *as query				

Cyanobacteria	152	3		

Firmicutes	68	1	1.33	0.64

Actinobacteria	120	3	0.8	0.76

Proteobacteria	166	5	0.67	0.82

*P. sojae*as query				

Cyanobacteria	151	4		

Firmicutes	60	2	0.79	0.77

Actinobacteria	94	1	2.6	0.37

Proteobacteria	163	3	1.4	0.46

*Thalassiosira*as query				

Cyanobacteria	232	31		

Firmicutes	51	0	NA	**0.003**

Actinobacteria	45	0	NA	**0.006**

Proteobacteria	160	2	10.3	**0.00002**

*Phaeodactylum *as query				

Cyanobacteria	199	32		

Firmicutes	45	1	7.3	0.014

Actinobacteria	62	1	10.1	**0.002**

Proteobacteria	162	4	6.44	**0.00003**

^1^Ratios are scaled to absolute number of first hits to avoid biases related to relative sizes of groups' genomic data sets.

^2^*P *values shown in bold are significant at experiment-wise a value of 0.05, adjusted to reflect multiple tests.

These results do not specifically address the efficacy of the chromalveolate model. The cumulative signal from diatom genomes reflects many genes related to plastid function that would have been lost from oomycete genomes along with the putative chromalveolate plastid. Moreover, even a strong signal from cyanobacterial genes has alternative possible explanations, such as prey biases in phagotrophic ancestors; therefore, we performed additional contingency tests on a more explicit prediction of the Chromalveolata.

According to the chromalveolate model, cyanobacterial genes that were not still retained in the plastid genome at the time of the secondary endosymbiosis (presumably the large majority had been lost from the plastid by that point) were passed to heterokonts through the red algal nucleus; note that genes still encoded in plastid genomes are not included in our analyses. We note that, based on this proposed history, it is unclear that putative cyanobacterial genes in oomycetes should be cited as evidence for chromalveolates at all; however, given sequence co-variation and the unusual nature of red genomes available, it appears reasonable that some genes transferred through the red nucleus now more closely resemble their cyanobacterial homologs. Nevertheless, if genes with a putative cyanobacterial ancestry are to be taken as evidence of the Chromalveolata, they generally should be more similar to red algal sequences than are genes from bacterial control groups. We compared the number of oomycete genes with first hits to each group that had red algae as the second hit, against those with "other than red algae" as the second hit. This also provided a correction for differences in total first hits associated with relative database size.

Contrary to *a priori *expectations of the chromalveolate model, cyanobacterial genes in oomycetes show no trend toward a greater affinity with red algae. In half of the comparisons, first hits to control groups show greater than expected proportions of red algal second hits, and in no case do differences approach statistical significance (Table 2). In contrast, putative cyanobacterial genes in diatom genomes show a dramatic proportional affinity to red algae against all control groups, and differences are significant in five of six cases (Table 2). The sixth case (*Phaeodactylum *genome, cyanobacteria versus firmicutes) favors a cyanobacterial-rhodophyte association at *P *= 0.014; however, this falls below experiment-wise significance after adjustment for multiple tests. Although a comparably strong signal is not expected in *Phytophthora*, which would have lost cyanobacterial genes related to photosynthetic function, there is no indication that cyanobacterial genes that were retained in oomycetes arrived via a red algal endosymbiosis. Thus, even if test results on total first hits (Table 1) are taken as evidence that there are more cyanobacterial genes in *Phytophthora *genomes than expected from a null model of sequence co-variation, the data are consistent with alternative evolutionary explanations rather than a chromalveolate origin [18,32].

### Red algal signal in heterokont genomes

To strengthen the correlation between BLASTP score and phylogenetic signal, we analyzed top hits to each eukaryotic group for which the next closest group had either a 5% or 10% weaker alignment score. As with the overall regressions on top hits (see below) of oomycete genes, those with potentially the strongest phylogenetic support were highly associated with size of data sets. Although hits to diatoms again deviate significantly from this trend, suggesting greater phylogenetic signal than predicted by the regression model, red algal sequences actually had lower than expected affinity (Table 3). Fisher exact tests, comparing first hits with proportionally strong similarity scores against total first hits, indicate that red algal sequences are no more represented in *Phytophthora *genomes than are those of the pre-assigned control group. To the contrary, in three of four comparisons there are proportionally more hits to amoebozoan than to red algal genes (Table 4). These results show that a red algal signal in the *Phytophthora *genomes does not deviate from the expectation from intragenomic co-variation from causes other than EGT under a "chromalveolate" model.

**Table 3:** Regression statistics for genes with proportionally high affinity to each defined group.

***Genome/Δ bit score to 2*^nd ^*hit*^1^**	**R^2^**	***P***	**Δ σ Heterokont**	**Δ σ Reds**
*P. ramorum/5%*	0.714	**0.007**	**2.71**	-0.12

*P. sojae*/5%	0.717	**0.007**	**2.67**	-0.15

*P. ramorum*/10%	0.629	0.019	**2.75**	-0.16

*P. sojae*/10%	0.644	0.016	**2.72**	-0.18

^1^Differences of 5% and 10% between bit scores to the top and second hits are used as a proxy for phylogenetic signal, based on demonstrated correlations between the two. Significant results are shown in bold.

**Table 4:** Fisher exact tests for a signal from red algal genes in oomycetes.

**Genome/Δ bit score for next hit**	**Reds**	**Amoebozoa**	**Ratio R:A^1^**	***P***
*P. ramorum*/5%	6	14	0.93	0.64

*P. sojae*/5%	3	13	0.67	0.81

*P. ramorum*/10%	1	1	2.2	0.53

*P. sojae*/10%	0	7	0	1.0

^1 ^Proportionally strong hits are scaled to absolute number of first hits, to adjust for bias from variation in sizes of databases

### Does the lack of cyanobacterial and red signals reflect loss of plastid-related genes?

To provide the most objective analysis of comparative signals in blastp hits to red algae relative to controls, we applied specific tests of the Chromalveolata using only those genes with significant hits to all six major eukaryotic taxa (see Additional file 1) included in the study. As much as possible, this minimizes biases from differential gene loss from small red algal genomes, as well as variation among genomes in individual or parallel cases of HGT from bacteria. It also removes potentially convergent or parallel signals associated with major metabolic processes that are differentially present among groups; for example, genes associated with flagella-based movement. Finally, and most importantly, it factors out those genes specifically related to plastid-function, which should not be found collectively in animal, fungal and amoebozoan genomes.

**Figure 2:**
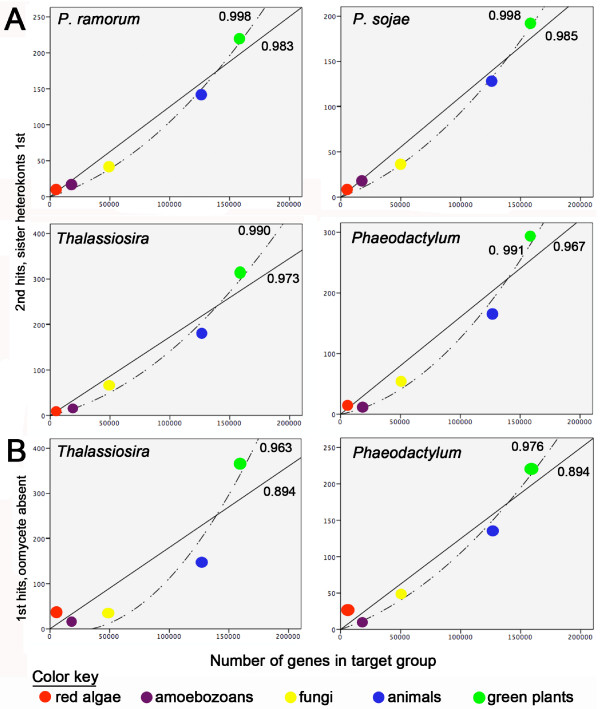
**Regressions showing red algal signal in diatoms is not shared with oomycetes**. A. Regressions on second hits for genes that are present in all eukaryotic groups (therefore, unrelated to plastid function), where the top hit is to the sister heterokont group (e.g. hit to oomycetes when diatoms are query sequences). The query genome in each case is shown in the upper right corner of the plot. Broken lines represent quadratic and solid lines linear regressions with adjacent R^2 ^values shown. In genes most similar between the heterokont sister groups, there is no apparent phylogenetic signal from red algae in either oomycete or diatom genomes; that is, hits to reds do not deviate positively from the value predicted by the regression model. B. Conversely, with oomycetes removed from the analysis, a regression on top hits versus group size clearly shows a positive signal for red algal genes. This same pattern was found in regressions on top hits against group size for all groups present (Additional file 2, Table 5). Contrary to predictions of the chromalveolate hypothesis, these comparative analyses indicate that the clearly detectable red algal signal in diatom genomes is not present in genes specifically shared with oomycetes.

Because the chromalveolate model predicts that most red algal genes already were present in the genome of the common ancestor of oomycetes and diatoms (Figure 1A), as an initial test for shared signal we performed analyses of second hits to each group when the known heterokont sister group is the first hit. Regressions of these hits on the size of database (Figure 2A) were highly significant, with R^2 ^values of nearly 1.0, and with no indication of a red algal deviation from the trend for *Phythophthora *genomes. Moreover, even the tendency for red algal hits to deviate positively from expected values in diatoms (Table 5, Additional file 2), all but disappears in genes specifically conserved between diatoms and oomycetes (Figure 2A). To investigate whether the lost red signal from diatoms could be hidden in genes not strongly conserved between diatoms and oomycetes, but nevertheless present in both taxa, we performed further analyses on diatoms genes with significant hits to all eukaryotic groups, but with oomycetes removed from the regressions. In this case, the positive deviation of red algal genes in diatom genomes once again becomes evident, although not significant in analyses of residuals (how much measured values deviate from those predicted by the regression) (Figure 2B, Table 5). Thus, in direct contrast to expectations from the chromalveolate model, the clearly detectable signal in diatoms from non-plastid-related genes of red algal origin is specifically absent from *Phytophthora *genomes.

**Table 5:** Regression statistics for total number of first hits versus size of database queried.

	**Analyses with all taxa**				**Analyses without heterokont sister group**		
**Genome**	**R^2^**	***P***	**No. σ^1^** **Heterokont**	**Δ σ Reds**	**R^2^**	***P***	**No. σ Reds**

*P. ramorum*^2^	0.841	**0.001**	**2.6**	-0.19	0.977	**<0.001**	0.363

*P. sojae*	0.855	**<0.001**	**2.46**	-0.047	0.965	**<0.001**	0.198

*Thalassiosira*	0.493	0.066	**2.71**	0.212	0.947	**<0.001**	1.597

*Phaeodactylum*	0.479	0.074	**2.71**	0.194	0.943	**<0.001**	1.515

^1 ^Residuals from regression analysis expressed in standard deviations (σ) from mean residual value. Significant results are in bold.

We performed additional and more explicit analyses of the relative signals from red algal genes in heterokont genomes, and whether they are shared between diatoms and oomycetes. Fisher exact tests on each group yielded mixed results for oomycetes. There is a significantly greater than expected proportion of first hits to red genes for the *P. ramorum *genome, once relative sizes of data sets are taken into account (Figure 1B). In contrast, there is no significant difference in hits to red algal versus control sequences for *P. sojae*.

As expected, the overall contributions of red algal genes to both diatom genomes are highly significant (Figure 1B). Because this remarkably significant signal in diatoms includes plastid-related genes, we performed additional Fisher exact tests on only those genes present in all eukaryotic groups (including animals, fungi and amoebozoans), effectively eliminating sequences associated with plastid function. There was a slight increase in detectable red algal signal in oomycete genomes, relative to total first hits, although it remained insignificant for *P. sojae *when adjusted for multiple tests (Figure 1C). Despite removal of plastid-related genes, however, the relative strength of red algal versus control signal in our positive control, diatom genomes, remained highly significant (Figure 1C).

To test whether red algal signals are shared between diatom and oomycete genomes, we repeated Fisher exact tests on those genes present in all eukaryotes that have top hits to the sister heterokont groups, and have either a red algal or control sequence as the second hit (Figure 1D). Remarkably, not only did the weak prevalence of hits to red algae disappear from the *P. ramorum *genome, so did all evidence for the extraordinarily significant impact of red algal sequences on diatom genomes. Diatom *P *values went from effectively 0.0 in comparisons of first hits to non-plastid-related genes, to effectively 1.0 when restricted to genes with clear similarity to oomycete homologs. Thus, virtually none of the pervasive signal in diatoms from the known red algal endosymbiosis is present in oomycete genomes and, most significantly, any red signal identified in either group is not shared between the two. This result is at odds with the most explicit prediction of the chromalveolate model (Figure 1A).

### Assessing the chromalveolate hypothesis

Our analyses give no indication that putative "algal genes" in oomycetes are remnants of the same rhodophyte-derived secondary plastid present in modern day ochrophytes. Quite the opposite, our results contradict specific predictions of the chromalveolate model. For example, although there is a weak tendency for *Phytophthora *genomes to yield a greater number of first hits to cyanobacterial genes than to two of three bacterial controls, there is no suggestion that this signal is associated with a red algal endosymbiosis (Tables 4, 5).

One potential complication of our analyses of cyanobacterial signal is the use of proteobacteria as a control group, given that the mitochondrion is believed to be derived from an α-proteobacterium-like ancestor [33]. This could result in a larger than expected number of first hits compared to other bacterial groups, including cyanobacteria. With respect to our specific results, however, we do not believe this is a significant concern. First, most mitochondria-derived proteobacterial genes present in oomycete nuclear genomes would have arrived via relatively ancient EGT, certainly before the divergence of oomycetes and ochrophytes. Thus, such genes conserved enough to identify homology by BLAST score should, as a rule, produce closer hits to a eukaryotic nuclear genome, the heterokont sister taxon in particular, than to anciently diverged proteobacterial genes. More importantly, in our explicit tests of the chromalveolate model, wherein rhodophyte-derived mitochondria-related genes would represent the most relevant complication, there is no evidence that mitochondrial EGT has biased proteobacterial signal relative to other bacterial control groups (Table 2).

Overall, there is no indication of any disproportionate contribution to *Phytophthora *genomes from a red algal endosymbiont, despite the highly significant signal in diatoms where endosymbiosis is known to have occurred. Moreover, the extraordinarily strong signal in diatoms is completely absent from genes specifically conserved in *Phytophthora*, and this difference clearly is not due to loss of plastid-related genes from oomycetes. Thus, even if putative cyanobacterial genes are taken as evidence of a concerted signal from historical HGT, they are better considered in light of alternative hypotheses to chromalveolates; for example, Doolittle's "you are what you eat" scenario [18], or the more ancient primary endosymbiosis proposed by Nozaki and colleagues [32,34].

Although pair-wise relationships between various "chromalveolate" constituent taxa have received support in some investigations, mostly in analyses of plastid-based characters, no evidence has been reported that supports the model as whole [7]. Moreover, plastid-related data suggesting relationships among select chromalveolate taxa are at least equally consistent with hypotheses of tertiary or serial endosymbioses [20,21,35,36]. In fact, despite a general consensus in plastid-based phylogenies, computational problems associated with investigations of complex and ancient evolution still leave open the possibility that red algal-derived plastids are not a monophyletic group [37], and that some plastidic chromalveolate taxa harbored green algae-derived plastids ancestrally [38,39]. It is important to note, however, that a monophyletic relationship among red-derived plastids, even if demonstrated unequivocally, supports only a common initial secondary origin, not their subsequent linear descent through the breadth of diversity comprising the Chromalveolata [21,35,36].

On the other hand, there is substantial evidence from sequence-based phylogenies that chromalveolate host cells do not represent a natural group [40-44], this despite potential impacts from EGT and EGR that should tend to draw them together in phylogenetic reconstructions even if they are unrelated [7,36]. Instead, photosynthetic chromalveolates are broken up by a number of heterotrophic groups, requiring numerous and complete plastid losses. There is a dearth of empirical data supporting such wholesale plastid loss in general [45], or for a photosynthetic history for most aplastidic chromalveolate taxa [7,36]. It is in this context that discovery of "algal" genes in heterotrophic or parasitic protists has taken on great importance as evidence of a lost secondary plastid. Our results argue strongly against such a preemptive interpretation in any aplastidic taxon.

Although our results provide rigorous statistical evidence against a chromalveolate model of plastid evolution, additional research is required to reject the Chromalveolata outright. It is particularly problematic that only the highly reduced and unusual red algal genomes currently available were used for complete genome-level analyses. It is also true, however, that investigations uncovering putative "algal genes" in aplastidic protists employ comparable genomic resources. In fact, we specifically targeted the same red data set used in the report by Tyler and colleagues [13], which has been cited extensively in support of the Chromalveolata. Thus, if unusual sequence evolution in available red genomes is deemed to make them unreliable for our statistical analyses, they also should be viewed as unreliable for inferring individual cases of EGT/EGR. In fact, unlike *a posteriori *interpretations of individual gene phylogenies, we employed *a priori *positive controls. They clearly show these unusual red genomes are sufficiently "normal" to demonstrate a highly significant contribution to diatom genomes, specifically in non-plastid-related genes. Thus, it is likely that gene content in oomycetes, rather than the peculiar nature of red genomes, is responsible for our results.

Overall, similarity signals from putative "algal" genes in oomycetes are more consistent with evolutionary co-variation that is unrelated to EGT or, if evidence of correlated HGT, they favor an alternative hypothesis to the Chromalveolata. In fact, given the general lack of support for chromalveolates as a natural group, even if we had found evidence for a plastid in the common ancestor of diatoms and oomycetes, it would not justify extending that result back to the ancestor of all "chromalveolate" taxa. In this case, however, we found no evidence for red EGT, even in the nearest available heterotrophic relatives of ochrophyte algae. This argues directly against the chromalveolate interpretation of plastid evolution.

### Association between genome similarity and size of data set

Our initial plan to compare red algal signal in heterokont genomes to multiple heterotrophic control groups (animals, fungi, amoebozoans) encountered a serious complication. With blastp results from all four heterokont genomes examined, both linear and quadratic regression models for number of first hits against size of targeted data set (total number of genes) were significant, and with very high values of R^2 ^(Table 5, Additional file 2).

Analyses of residuals demonstrate that, for BLASTP querying with each *Phytophthora *genome, only hits to diatom genomes deviate significantly (>2 σ) from the predicted number of top hits based solely on an association with total gene number (Table 5). The large positive deviation for diatoms is consistent with their close phylogenetic relationship to oomycetes; however, for no other group does the deviation approach significance. This suggests that, 1) different evolutionary trajectories of oomycetes and diatoms have not erased phylogenetic similarity in many orthologous genes, and 2) artifacts related to database size tend to swamp cumulative signal from phylogenetic relatedness, as measured by overall sequence similarity, except at relatively close evolutionary distances.

The association between BLAST similarity and database size is demonstrated even more clearly when respective heterokont sister groups are removed from the regression. This results in highly significant correlations with R^2 ^values greater than 0.94 for all four genomes (Table 5, Additional file 2). Initially, we hypothesized that this trend could simply reflect greater gene loss from groups with fewer and smaller available genomes. As noted above, however, calculated regressions on genes returning significant hits to all six eukaryotic groups also were highly significant. Therefore, variation in gene loss cannot account for our results. This was true even with sequences retaining enough phylogenetic signal so that a sister heterokont genome produced the first hit (Figure 2A). In fact, rather than providing at least some correction for possible bias from gene loss, significance of the correlation and R^2 ^values actually increased substantially.

This strong correlation was not fully overcome by signal from EGT, even when a known endosymbiosis has occurred. For queries with diatom genes, regressions of first hits on database size were not significant when all groups were included in the analyses (Table 5). In analyses of residuals, the known sister group (oomycetes) deviates significantly from expected values for all four heterokont genomes. Unlike analyses of oomycetes, however, red algal genes also deviate in a positive direction in diatom genomes, although not significantly. With oomycetes removed, regressions on diatoms became highly significant but the relative magnitude of positive deviation of red algae also increased substantially, albeit not significantly at >2 σ, (Table 5; Figure 1). These data suggest that combined phylogenetic signal from oomycete and red algal genes is responsible for the absence of a significant correlation in full analyses of first hits querying with diatom genomes (Table 5).

**Figure 3:**
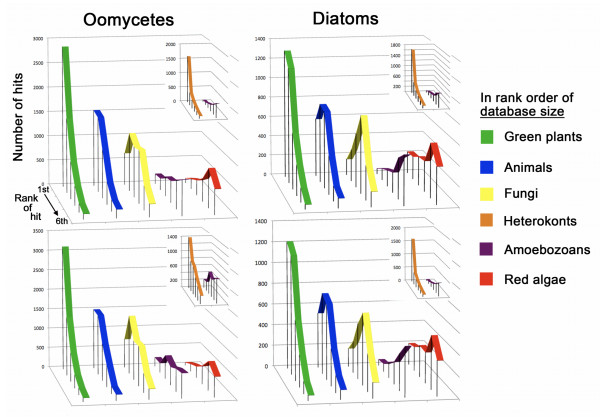
**Distributions of all hits from eukaryotic groups to each heterokont genome**. Number of times each designated group is the first to be hit in BLAST searches, through the number of times it yields the sixth hit, are shown from back to front of each graph. Clockwise from the upper left, panels show *P. ramorum*, *Thalassiosira*, *Phaeodactylum *and *P. sojae*. In each case, defined groups appear in rank order of database size from left to right. The exception is the sister heterokont group, which is shown inset and next to amoebozoans, the unrelated control group with the nearest-sized database.

More unexpected was the result that biases related to the number of genes from each group extend beyond counts of total or first hits. There also is a clear trend in the distribution of hits to each respective target group. The larger the size of the subject data set, the more hits are skewed in the direction of greater similarity to the query gene (Figure 3). The only group to deviate from this trend is the heterokont sister taxon (see insets on Figure 3). Perhaps most intriguing, with respect to potential effects on *a posteriori *hypotheses development, the shapes of the distributions of ranked hits to green plants (largest data set) and animals (second largest) are similar to those recovered from heterokont sister genomes of known evolutionary affinity. That is, the patterns from these divergent and unrelated taxa effectively reproduce similarity distributions that result from a close phylogenetic relationship, potentially based only on the relatively large sizes of the genome databases sampled. The distribution shape for fungi, the next largest data set, also is skewed toward greater proportional similarity relative to red algae and amoebozoans. This is why we used only amoebozoans as a control group in Fisher exact tests of the chromalveolate model, to reduce collective biases related to database size as much as possible.

### Broader considerations of *a posteriori *evidence of endosymbiotic gene transfer

Our results indicate that caution should be exercised when interpreting the presence of genes consistent with EGT, especially as database sizes for particularly well-studied taxa continue to grow disproportionately. In light of the large number of highly similar hits to each of our designated groups, it is likely that a number of hypothetical *a posteriori *scenarios of gene transfer could be inferred from these genomes. For example, biased similarity distributions in potentially phylogenetically informative genes (Table 3, Figure 3) undoubtedly could be used to generate strong *a posteriori *evidence of concerted HGT for groups like green plants and animals; both have large numbers of sequences showing disproportionately strong similarities to heterokont homologs. There is no basis to conclude that detailed phylogenetic analyses of all candidate genes, as typically carried out when searching for individual examples of EGT [12], would distinguish between bias-related artefacts and *bona fide *examples of HGT.

Anecdotally, we had no trouble finding trees that strongly support a phylogenetic association between oomycete genes and sequences from each of the major groups in our investigation. More significantly, Maruyama and colleagues [46] recently performed genome-level screens of various protists, using a BLAST plus phylogenetic analysis pipeline, and found a comparable number of "algal" genes in the heterolobosean amoeba *Naeglaria *and choanoflagellate *Monosiga *as in oomycetes. Although the results from *Naeglaria *could be explained by a more ancient primary endosymbiosis [32], no current model of plastid evolution predicts EGT into *Monosiga*, a member of the opisthokonts. This study demonstrates that direct phylogenetic evidence for EGT in oomycetes is comparable to that found in taxa that are, effectively, aplastidic "controls" under the assumptions of the chromalveolate model. This adds strong support to our argument that pre-assigned and careful controls are necessary to verify support for correlated HGT or EGT over alternative explanations of persistent phylogenetic incongruence.

## Conclusion

Whole genomes comprise large, complex data sets, with apparent phylogenetic conflicts among sequences stemming from a variety of known and unknown factors [2]; horizontal gene transfer is only one of them. The strong associations we uncovered, between trends in sequence similarity and number of genes queried, suggest that co-variation in genome size adds yet another factor to be considered in phylogenomic analyses. For all these reasons, gene candidates for EGT retrieved from genome-level surveys of aplastidic taxa should not simply indicate a strong phylogenetic affinity to one or another algal group. They also should be subjected to tests against null and alternative hypotheses, each with different and specific *a priori *expectations, before they are interpreted as evidence for any particular model of EGT. In the specific case of *Phytophthora *genomes, our results indicate null or alternative models are favored over the chromalveolate hypothesis.

Co-variation in genome size is one of a number of recognized sources of directional and stochastic bias that can produce strong artefacts in phylogenomic investigations [2,15,47]. This uncharacterized sequence co-variation provides a fertile hunting ground for individual or handfuls of cases that support one or another evolutionary scenario, even if an alternative hypothesis is favored by comparable or total evidence. Given the ever-increasing amount and complexity of data available for phylogenomic investigations, it appears prudent to begin to move away from *a posteriori *data interpretations, and toward direct tests of explicit predictions from standing and future evolutionary hypothesis.

## Methods

### Database creation and sequence similarity searches

To investigate signal from algal sequences in *Phytophthora *genomes, two protein sequence databases were created. BLASTP searches were performed using predicted protein sequences of *P. sojae *and *P. ramorum *as queries against a database comprising a range of eukaryotic and bacterial genomes (DB1, see below). Because of the existence of a red algal derived plastid in ochrophytes, we also queried a second database (DB2) using protein sequences of diatoms *Thalassiosira pseudonana *and *Phaeodactylum tricornutum*. Searches with diatom sequences served as a positive control, to determine the strength of signal when a red endosymbiosis is known to have occurred. Because our focus was on the identification of the most similar sequences and reliable homologs from the databases, a stringent cutoff E-value 1e-20 was used in all searches.

Predicted protein sequence data for *P. sojae *and *P. ramorum *were downloaded from the genome sequencing projects of the two species at the Joint Genome Institute. All other sequences were obtained from NCBI genome database and corresponding genome sequencing projects. These databases cover genomes sampled in Tyler et al. [13] and additional sequences from several other eukaryotic and bacterial groups. Specifically, the first database (DB1) includes predicted protein sequences from fungi (*Saccharomyces cerevisiae*, *Schizosaccharomyces pombe*, *Magnaporthe grisea*, *Neurospora crassa*, *Aspergillus fumigatus*, and *Ustilago maydis*), animals + Choanozoa (*Monosiga brevicollis*, *Homo sapiens*, *Mus musculus*, *Drosophila melanogaster*, and *Caenorhabditis elegans*), diatoms (*T. pseudonana *and *P. tricornutum*), red algae (*Cyanidioschyzon merolae*, *Guillardia theta *nucleomorph), green plants (*Ostreococcus tauri*, *Chlamydomonas reinhardtii*, *Physcomitrella patens*, *Arabidopsis thaliana*, and *Oryza sativa*), amoebozoa (*Dictyostelium discoideum*, *Entamoeba histolytica*), cyanobacteria (*Crocosphaera watsonii *WH 8501, *Microcystis aeruginosa *NIES-843, *Anabaena variabilis *ATCC 29413, *Gloeobacter violaceus *PCC 7421, *Prochlorococcus marinus *MIT 9313, *Synechococcus elongatus *PCC 6301, *Synechocystis sp*. PCC 6803, *Trichodesmium erythraeum *IMS101, *Lyngbya aestuarii *CCY9616, *Acaryochloris marina *MBIC11017), firmicutes (*Bacillus subtilis subsp. subtilis *str. 168, *Staphylococcus aureus subsp. aureus *JH1, *Lactobacillus reuteri *F275, *Clostridium perfringens *str. 13, *Enterococcus faecalis *V583, *Leuconostoc mesenteroides subsp. mesenteroides *ATCC 8293, *Desulfitobacterium hafniense *Y51, *Thermoanaerobacter pseudethanolicus *ATCC 33223, *Acholeplasma laidlawii *PG-8A, and *Carboxydothermus hydrogenoformans *Z-2901), actinobacteria (*Streptomyces avermitilis *MA-4680, *Frankia sp*. EAN1pec, *Bifidobacterium longum *NCC2705, *Acidothermus cellulolyticus *11B, *Salinispora arenicola *CNS-205, *Nocardia farcinica *IFM 10152, *Thermobifida fusca *YX, *Arthrobacter aurescens *TC1, *Mycobacterium marinum *M, *Propionibacterium acnes *KPA171202), and proteobacteria (*Acidiphilium cryptum *JF-5, *Aeromonas hydrophila subsp. hydrophila *ATCC 7966, *Chromobacterium violaceum *ATCC 12472, *Geobacter uraniumreducens *Rf4, *Marinobacter aquaeolei *VT8, *Nitrosococcus oceani *ATCC 19707, *Photobacterium profundum *SS9, *Ralstonia eutropha *H16, *Rhodobacter sphaeroides *ATCC 17029, and *Rhodospirillum rubrum *ATCC 11170). The second protein sequence database (DB2) was identical to DB1 except that diatom sequences (*T. pseudonana *and *P. tricornutum*) were replaced by those of the two *Phytophthora *species.

### Data processing and computation

To perform computational sorting of BLASTP data, first we developed a C++ program to parse the BLAST output files and extract the relevant information for each given statistical analysis. We then created a database using MySQL to store the information and performed various further computations, including data sorting and counting, using PHP and MySQL. Our implementation platform was Windows XP/Dev C++4.9.9.2/Apache Web Server 2.2/MySQL 5.0/PHP5.2.5.

The C++ program we developed used the genome-wide BLASTP output file as input, and generated two new output files. The first output file stored information about the top hits in the following format:

Input sequence/similar sequence found/hit#/similar sequence/group ID/group name/genome/score/e-value, where "similar sequence found" indicates whether a sequence above the threshold value was returned for the each given gene from the heterokont genome used to query target groups. The "group" refers to each of the ten designated target taxa described above (also see Additional file 1). Group information, such as ID, group name, and genome were placed in a separate file so that any changes made to the group information file had no effect on the computational program. This permitted multiple alternative runs using the same basic sorting algorithm. The second output file stored more detailed information about first hits, second hits, etc. in the following example format:

Group ID/group name/input sequence/hit #1 genome/hit #1 e-value/hit #2 group ID/hit #2 group name/hit #2 e-value/hit #1 score/hit #1 similar sequence/hit #2 score.

We used these programs to sort four datasets created from BLASTP searches. Each dataset generated two output files as described above. Based on these two output files, we created a database with 11 tables to store all the information. Additional PHP scripts were developed to perform counting and sorting of this output database as discussed in our results.

### Statistical analyses

Our initial BLASTP query using the *P. ramorum *genome yielded a clearly observable association between the number of top hits retrieved from target groups and the size of their databases (in number of sequences present). To investigate this further, we performed linear and quadratic regressions in SPSS (version 16.0) with forced zero intercepts. The number of BLASTP hits returned was regressed on the number of genes in each group's database in separate queries with each heterokont genome. These analyses were performed using different taxonomic groups and genes as follows: with all groups present, with the sister heterokont removed, on only genes present in all eukaryotic genomes, for genes present in all eukaryotes where the top hit had a 5% or 10% higher bit score than the second hit, and on genes present in all eukaryotic genomes for which the sister heterokont produced the top hit. To identify taxa that deviate significantly from the observed relationship, residuals were calculated and expressed as standard deviations from the mean residual value. Because we had no prior expectation for the observed correlations, and quadratic regressions generally yielded slightly higher R^2 ^values and equal or greater significance levels, analyses of residuals were performed based on quadratic models.

Contingency analyses (Fisher exact tests) were carried out using on-line software [48] to test specific predictions of the chromalveolate model. In each case variables were chosen to best correct for potential biases caused by associations between numbers of BLAST hits and relative databases sizes. Tests were as follows. 1) Hits to cyanobacteria should show greater affinity to red algal genomes than do hits to bacterial control groups. Variables were the top hits to cyanobacteria or to members of each control group, when red algae was the second group hit, versus number of top hits when other than red algae was the second group hit. 2) There should be more first hits to red algae than to eukaryotic control groups with no phylogenetic or endosymbiotic relationship to heterokonts. To correct for association biases, amoebozoans were chosen as the specific control group because they were closest to red algae in both total number of genes in the database and the ranked distributions of hits (Figure 3). Variables were total first hits to each of the two databases versus total hits that were not first. This analysis was repeated using only those genes present in all eukaryotic groups, to factor out genes specifically related to plastid-function in diatoms and would, therefore, likely have been lost from oomycetes (were they secondarily aplastidic). 3) To test the prediction that a red algal signal should be shared between oomycetes and diatoms, variables were second hits to both red algae and amoebozoans when first hits were to the other heterokont group, versus second hits when the first hit was to other than the sister heterokont. One-tailed P values are reported based on expectations of the chromalveolate model, with significance at P = 0.05 adjusted for multiple tests as appropriate [20].

## List of Abbreviations

EGR: endosymbiotic gene replacement; EGT: endosymbiotic gene transfer; HGT: horizontal gene transfer.

## Authors' contributions

JWS helped to conceive and design the study, worked on statistical analyses and was primary author of the manuscript. JH helped to conceive and design the study, assembled genome databases and performed similarity searches. QD and JT designed and wrote scripts for computational sorting of BLAST results. CG designed and worked on statistical analyses. All authors worked on, read and approved the final manuscript.

## Supplementary Material

Additional file 1 **Table of genomes analyzed**. Complete list of the genomes queried and their sizes in total number of annotated genes present. Click here for file

Additional file 2 **Regression results on total first hits**. Figure showing total first hits to each target group regressed against database size, querying with all four heterokont genomes. Click here for file
