# Supplementary material for: Are algal genes in nonphotosynthetic protists evidence of historical plastid endosymbioses?
Source: BMC Genomics. 2009 Oct 20;10:484. doi: 10.1186/1471-2164-10-484 (PMC2770532; doi:10.1186/1471-2164-10-484)
Supplement: Additional file 1 — Table of genomes analyzed. Complete list of the genomes queried and their sizes in total number of annotated genes present. [file 1471-2164-10-484-S1.PDF]

Additional file 1 – Genomes queried

Red algae (2)
Cyanidioschyzon merolae
Guillardia theta
Total: 5258 sequences

Green plants and green algal relatives (5)
Arabidopsis thaliana
Oryza sativa
Chlamydomonas reinhardtii
Physcomitrella patens
Ostreococcus tauri
Total: 158454 sequences

Diatoms (2)
Phaeodactylum tricornutum
Thalassiosira pseudonana
Total: 22415 sequences

Oomycetes (2)
Phytophthora ramorum
Phytophthora sojae
Total: 34770 sequences

Animals and choanoflagellate relative (5)
Homo sapiens
Mus musculus
Drosophila melanogaster
Caenorhabditis elegans
Monosiga brevicollis
Total: 126648 sequences

Fungi (6)
Sacchromyes cerevisiae
Schizosaccharomyce pombes
Magnaporthe grisea
Neurosphora crassa
Aspergillus fumigatus
Ustilago maydis
Total: 49948 sequences

Amoebozoa (2)
Dictyostelium discoideum
Entamoeba histolytica
Total: 18009 sequences

Cyanobacteria (10)
Crocosphaera watsonii NIES-843
Microcystis aeruginosa
Anabaena variabilis ATCC 29413
Gloeobacter violaceus PCC 7421
Prochlorococcus marinus str. MIT 9313
Synechococcus elongatus PCC 6301
Synechocystis sp. PCC 6803
Lyngbya aestuarii CCY9616
Trichodesmium erythraeum IMS101
Acaryochloris marina MBIC11017
Total: 49711 sequences

Firmicutes (10)
Bacillus substilis subsp. subtilis str. 168
Staphylococcus aureus subsp. aureus JH1
Lactobacillus reuteri F275
Clostridium perfringens str. 13
Enterococcus faecalis V583
Leuconostoc mesenteroides subsp. mesenteroides ATCC 8293
Desulfitobacterium hafniense Y51
Thermoanaerobacter pseudethanolicus ATCC 33223
Acholeplasma laidlawii PG_8A
Carboxydothermus hydrogenoformans Z-2901
Total: 28081 sequences

Actinobacteria (10)
Streptomyces avermitilis MA-4680
Frankia sp. EAN1pec
Bifidobacterium longum NCC2705
Acidothermus cellulolyticus 11B
Salinispora arenicola CNS-205
Nocardia farcinica IFM 10152
Thermobifida fusca YX
Arthrobacter aurescens TC1
Mycobacterium marinum M
Propionibacterium acnes KPA171202
Total: 44506 sequences

Proteobacteria (10)
Acidiphilium cryptum JF-5
Aeromonas hydrophila subsp. hydrophila ATCC 7966
Chromobacterium violaceum
Geobacter uraniumreducens Rf4
Marinobacter aquaeolei VT8
Nitrosococcus oceani ATCC 19707
Photobacterium profundum SS9
Ralstonia eutropha H16
Rhodobacter sphaeroides ATCC 17029
Rhodospirillum rubrum ATCC 11170
Total: 43822 sequences
