# Supplementary material for: Are algal genes in nonphotosynthetic protists evidence of historical plastid endosymbioses?
Source: BMC Genomics. 2009 Oct 20;10:484. doi: 10.1186/1471-2164-10-484 (PMC2770532; doi:10.1186/1471-2164-10-484)
Supplement: Additional file 2 — Regression results on total first hits. Figure showing total first hits to each target group regressed against database size, querying with all four heterokont genomes. [file 1471-2164-10-484-S2.PDF]

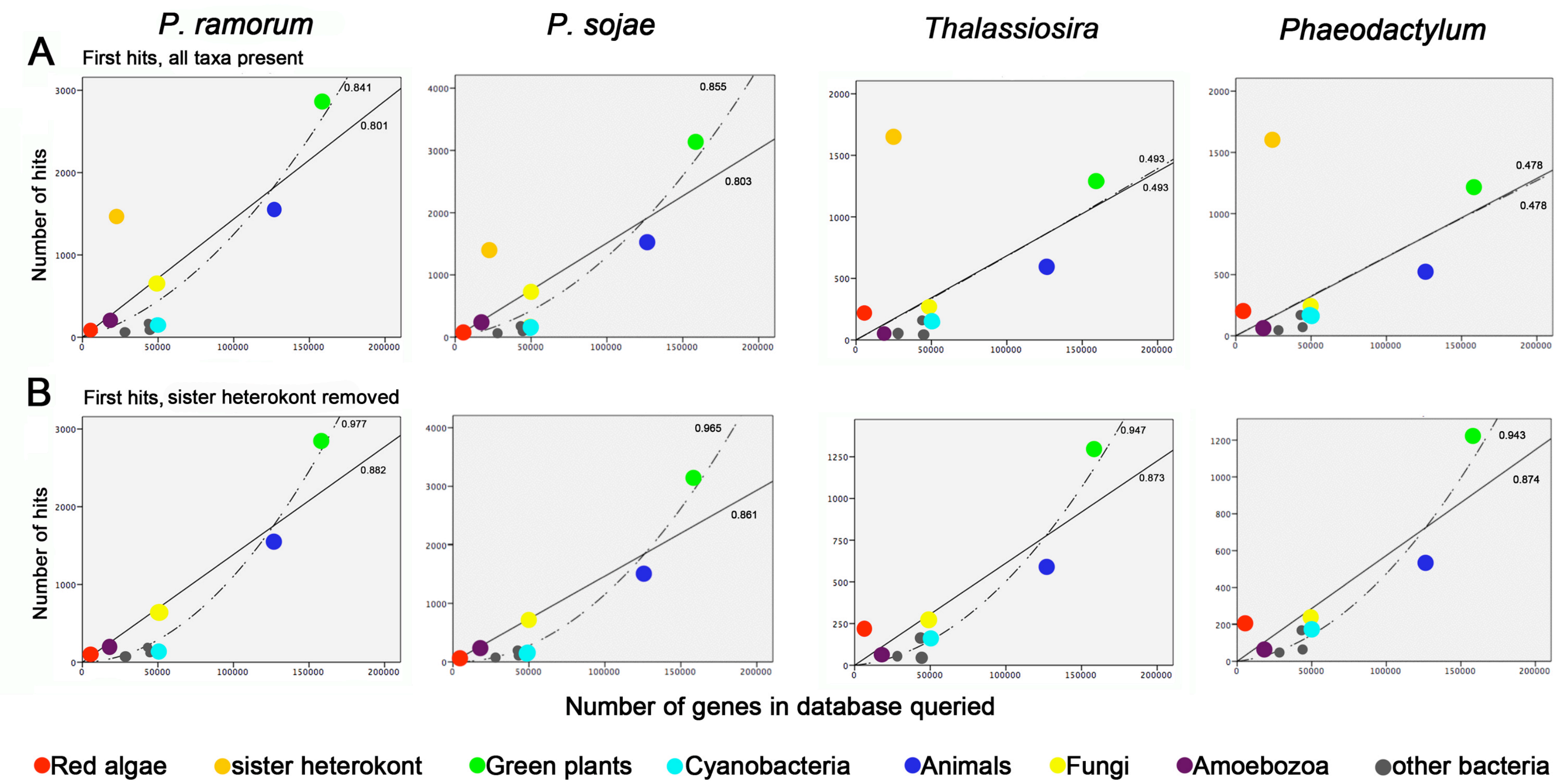

Additional file 2. Regressions on all first hits to each target group against database size, querying with all four heterokont genomes. Solid lines are linear and dashed lines are quadratic regression models. Numbers adjacent to each line provide R^2 values for each model. In all cases there is a clear positive deviation from the expectation for hits to the sister heterokont taxa (hits to diatoms when querying with oomycetes, hits to oomyctes when querying with diatoms). Analyses of residuals show that the positive deviation of the sister heterokont is significant in all cases (see Table 5), demonstrating that overall BLAST similarities do provide a robust measure of relatedness at closer evolutionary distances. There are no indications of a positive deviation in hits to red genes for oomycete genomes. The red algal positive deviation, particularly with the oomycetes removed, is observable in diatoms genomes but does not reach statistical significance.
